# First-line treatment of chronic hepatitis B with entecavir or tenofovir in ‘real-life’ settings: from clinical trials to clinical practice

**DOI:** 10.1111/j.1365-2893.2012.01602.x

**Published:** 2012-06

**Authors:** S Pol, P Lampertico

**Affiliations:** 1 Unité d’Hépatologie, Hôpital Cochin, Université Paris DescartesAPHP, INSERM U.1016, Paris, France; 2 First Gastroenterology Unit, Fondazione IRCCS Ca’ Granda Ospedale Maggiore Policlinico Università di MilanoMilan, Italy

**Keywords:** entecavir, hepatitis B virus, nucleoside/nucleotide analogues, tenofovir

## Abstract

Entecavir (ETV) and tenofovir disoproxil fumarate (TDF) are potent nucleos(t)ide analogues (NUCs) recommended as first-line monotherapies for chronic hepatitis B. In Phase III trials, ETV and TDF demonstrated superior efficacy, and comparable safety compared with other NUCs. In long-term clinical studies, both drugs achieved virologic response rates of around 95%, with very low rates of resistance development and good safety profiles. Clinical trials are conducted under standardized conditions with strict enrolment criteria that limit the heterogeneity of study populations. ‘Real-life’ populations tend to be composed of a wider range of patients, often older and with different morbidities, comorbidities that may impact treatment efficacy and co-factors, such as smoking and alcohol intake, which can have a direct impact on disease progression. Real-life studies provide better representations of everyday clinical practice and are important to confirm the results reported in clinical studies and to identify rare or late-emerging adverse events. In five ‘real-life’ studies of ETV in more than 1000 patients, up to 4 years of treatment resulted in virologic responses in 76–96% of patients. Two real-life studies of TDF reported response rates of 71–92% after up to 21 months of treatment. Low incidences of drug resistance and favourable tolerabilities were reported for both drugs, thus confirming the results from registration trials.

## Introduction

It is estimated that one-third of the world’s population has serologic evidence of a past or present hepatitis B virus (HBV) infection, with around 370 million being chronically infected [1,2]. HBV-related liver failure, cirrhosis and hepatocellular carcinoma (HCC) currently account for over 1 million deaths annually [2]. The goal of chronic hepatitis B (CHB) treatment is to improve survival by preventing disease progression to decompensated cirrhosis and HCC [1–3]. It is now well established that the risk of disease progression is reduced through the sustained reduction in HBV DNA to undetectable levels [4–6]. The effective and sustained suppression of HBV replication can result in regression of liver fibrosis and can even reverse liver cirrhosis [7]. Furthermore, maintaining undetectable levels of HBV DNA also increases the rate of hepatitis B e antigen (HBeAg) and hepatitis B surface antigen (HBsAg) seroconversion, which are also desired endpoints of CHB therapy [1,2]. However, HBV is not completely eradicated by treatment, even if HBsAg loss occurs. This is because of the persistence of nuclear covalently closed circular DNA and HBV DNA integrated into the host genome, which may trigger HBV reactivation or direct viral hepatocarcinogenesis, respectively. Long-term therapy is required in HBeAg(−) and in HBeAg(+) patients who cannot maintain virologic suppression off-treatment and for those with advanced liver disease. One barrier to the success of long-term therapy is the emergence of drug-resistant mutants, which are frequently observed during treatment of CHB with lamivudine (LVD), adefovir (ADV) or telbivudine as monotherapies [2]. Current guidelines, therefore, recommend that the most potent drugs with optimal resistance profiles (i.e. entecavir [ETV] and tenofovir disoproxil fumarate [TDF]) should be used as first-line monotherapies in CHB [2,3]. These two agents were approved by the US Food and Drug Administration (FDA) for the treatment of CHB on the basis of Phase III clinical study results in 2005 (ETV) and 2008 (TDF). Since that time, accumulating data from observational ‘real-life’ cohort studies have added considerably to our understanding of the efficacy and safety profiles of these two drugs. This review aims to summarize the currently available clinical practice or ‘real-life’ data for ETV and TDF as first-line treatments for CHB.

## The Importance of Clinical Trial and Clinical Practice Data

The initial evaluation and approval of any new drug is based on Phase III clinical trials. These are necessary to clearly establish the efficacy and safety of the drug in comparison with the current standard of care, in a controlled setting. Phase III clinical trials are conducted on large patient groups under standardized conditions with strict enrolment criteria, generally eliminating older patients and those with co-infections or comorbidities (such as cirrhosis) that could complicate the analysis of the results. Centralized monitoring of efficacy endpoints is carried out at regular intervals for all patients, using standardized protocols, and is generally more extensive than would be usual in clinical practice. This allows for rigorous assessment of antiviral potency, safety, resistance and histology, which is difficult to assess in a controlled manner in a real-life clinical practice setting.

In Phase III clinical studies, ETV proved to be superior to LVD in terms of virologic, biochemical and histologic outcomes in both HBeAg(+) and HBeAg(−) nucleos(t)ide analogue (NUC)-naïve CHB patients [8,9]. In the subsequent rollover study ETV-901, long-term therapy with ETV resulted in durable and increasing viral suppression, with undetectable levels of HBV DNA (<300 copies/mL) being achieved by 94% of HBeAg(+) patients over 5 years of treatment, and in 95% of HBeAg(−) patients over 3 years of treatment [10,11]. In HBeAg(+) patients, continued virologic suppression leads to increasing serologic responses; over 96 weeks of treatment, 31% (110/354) and 5% (18/354) of patients achieved HBeAg seroconversion and HBsAg loss, respectively. Continued treatment of those who remained HBeAg(+) at week 96 resulted in HBeAg seroconversion and HBsAg loss in 33/141 (23%) and 2/145 (1.4%) additional patients, respectively [12]. Results also demonstrated that ETV was well tolerated [13] and that the emergence of viral resistance remained minimal (1.2%) following up to 6 years of treatment [14,15]. Importantly, histologic analyses of liver biopsies from 57 patients receiving ETV for a median of 6 years (range 3–7 years) revealed that 96% of patients demonstrated histologic improvement (a decrease by at least 2 points in the Knodell necroinflammatory score, without worsening of fibrosis) and 88% had reduced fibrosis (a decrease of at least 1 point in the Ishak fibrosis score) [7]. Reversal of advanced fibrosis/biopsy-proven cirrhosis was demonstrated in nine of 10 patients with baseline Ishak fibrosis scores of 4–6 who underwent serial liver biopsies up to year 6.

Similarly, in Phase III studies, TDF has demonstrated superior antiviral efficacy over ADV in HBeAg(+) and HBeAg(−) CHB patients [16]. Durable and increasing viral suppression was observed over 4 years of treatment with undetectable HBV DNA (<400 copies/mL), achieved by 96% of HBeAg(+) patients and 99% of HBeAg(−) patients [17,18]. In HBeAg(+) patients, HBeAg loss occurred in 41% of patients and HBeAg seroconversion in 29%; the cumulative probability of HBsAg loss was 11%. TDF was well tolerated over this treatment period [17,18], and no resistance has been reported to date. 5% of the patients without complete viral suppression (HBV DNA >400 copies/mL) treated over this time period switched to TDF in combination with emtricitabine at or after week 72 [19,20]. As with ETV, long-term treatment with TDF has been associated with histologic improvement. A recent report demonstrated sustained viral suppression over 5 years of TDF treatment was concomitant with histologic improvement and significant biopsy-proven regression of fibrosis and cirrhosis in 226 patients [21].

These results clearly demonstrate the efficacy and safety of ETV and TDF in the controlled environment of randomized clinical studies. However, there are many differences between patients included in registration studies and those encountered in everyday life. Real-life populations tend to be more heterogeneous, having a broader age range and including patients with various comorbidities such as diabetes, renal impairment and obesity. Co-factors such as smoking and alcohol intake, which can have a direct impact on fibrosis progression, may also be more common, and patients outside of a clinical study setting may be less likely to maintain good treatment adherence. Real-life data are, therefore, required to confirm the efficacy and tolerability data reported in clinical studies and to continue safety monitoring in order to identify rare or late-emerging adverse events.

## Efficacy of Entecavir in Real-Life Settings

Six real-life studies assessing the efficacy and safety of ETV in a total of 1296 NUC-naïve patients have been presented or published, all confirming results reported in clinical trials, with similar rates of virologic and serologic responses and a low incidence of resistance. Importantly, these studies contain a heterogeneous mixture of patients who are differentiated from those in clinical trials as based on a number of criteria (Table 1) and may, therefore, be more reflective of the treatment population and the real efficacy and safety of the drug. Results from these real-life studies are discussed in the following sections and summarized in Table 2.

**Table 1:** Baseline characteristics of patients included in entecavir (ETV) studies

Characteristic, *n* (%)*	ETV-022	ETV-027	ORIENTE	VIRGIL	Argentinean cohort	King’s College Cohort	Italian cohort	Hong Kong cohort
Reference	[9]	[10]	[22]	[23]	[25]	[26]	[27]	[28]
*N*	354	325	190	243	69	154	418	222
Age, years (SD or range)	35 (13)†	44 (11)†	44 (35–54)‡	43 (14)†	46 (11)†	42‡	58 (18–82)§	47 (21–77)§
Male	274 (77)	248 (76)	139 (73)	177 (73)	51 (74)	122 (79)	316 (76)	157 (71)
Race								
White	140 (40)	193 (59)	60 (84)¶	114 (47)	60 (87)	NR	NR	NR
Asian	204 (58)	122 (38)	18 (9)	70 (29)	9 (13)			
Black	8 (2)	8 (2)	6 (3)	NR				
Other	2 (<1)	2 (<1)	6 (3)	59 (24)				
Region								
Europe	84 (24)	156 (48)	Spain	Europe	Argentina	UK	Italy	Hong Kong
North America	47 (13)	28 (9)						
South America	51 (14)	35 (11)						
Australia and Asia	172 (49)	106 (33)						
Genotype D	37 (10)	157 (48)	NR	91 (50)	NR	NR	84/93 (90)	NR
HBeAg(−)	6 (3)	322 (99)	133 (70)	157 (65)	25 (36)	106 (69)	347 (83)	132 (59)
HBV DNA, log_10_ IU/mL*	9.62 (2.01)†,¶	7.6 (1.8)†,¶	5.94 (4.64–7.39)‡	6.2 ± 1.7‡	7.09 (1.85)†	4.6 (0.2) **	6.0 (1.5–9)§	7.1 (4.0–>8.8)§
ALT, IU/L*	140.5 (114.3)†	141 (114.7)†	71.5 (44–108)‡	NR	157 (211)†	NR	92 (11–2241)§	92 (17–2168)§
Cirrhosis	26/329 (8)	15/303 (5)	0††	57 (24)	16 (11)	52 (34)	202 (49)	0

ALT, alanine transaminase; NR, not reported; NUC, nucleos(t)ide analogue; VIRGIL, Vigilance against Viral Resistance.

* Unless otherwise specified.

† Mean (standard deviation).

‡ Median (interquartile range).

§ Median (range).

¶ log_10_ copies/mL.

** Mean (standard error).

†† Advanced fibrosis in 34%.

**Table 2:** Summary of efficacy results from real-life studies of entecavir in NUC-naïve patients

Study [ref]	Median follow-up (range)	No. patients	Cut-off (assay limit) (IU/mL)	HBV DNA undetectable, % (*n*/N*)	ALT normalization, % (*n*/N*)†	HBeAg seroconversion, % (*n*/N*)‡	HBsAg loss, % (*n*/N*)
ORIENTE [22]	52 weeks (46–53)	190	50	83 (150/181)	82 (115/141)	21 (12/57)	1 (2/190)
VIRGIL [23]	19 months (3–45)	243	80	86 (208/243)	74 (126/171)	15 (13/86)	1 (3/243)
Argentinean cohort [25]	110 weeks (56–164)	69	6	88 (61/69)	98 (63/64)	44 (19/43)	10 (7/69)
King’s College cohort [26]	28 months (NR)	154	12	76 (NR)	NR	8 (NR)	1 (NR)
Italian cohort [27]	42 months (2–53)	418	12	99 (66/67)	88 (60/68)	56§ (27 patients)	21§ (12 patients)
Hong Kong cohort [28]	3 years (12–60 months)	222	12	96 (67/70)	90 (51/57)	53 (16/30)	0.5 (1/222)§

ALT, alanine transaminase; HBeAg, e antigen; HBsAg, surface antigen; HBV, hepatitis B virus; NR, not reported; NUC, nucleos(t)ide analogue; VIRGIL, Vigilance against Viral Resistance.

* N = number of patients on treatment, unless stated otherwise.

† Among those with elevated ALT at baseline.

‡ Among those HBeAg(+) at baseline.

§ Kaplan–Meier estimate.

### The ORIENTE study

Results from a retrospective multicentre study conducted at over 25 Spanish centres were presented at the 2010 American Association for the Study of Liver Diseases (AASLD) meeting [22]. The ORIENTE study evaluated 190 mostly HBeAg(−) patients with compensated liver disease who were treated with ETV (0.5 mg) over a 1-year period. Findings were consistent with those observed in Phase III clinical studies over the same time period, with the rate of virologic response (undetectable HBV DNA) being achieved in 61% and 92% of HBeAg(+) and HBeAg(−) CHB patients, respectively, with 21% (12/57) achieving HBeAg seroconversion and 1% (2/190) clearing HBsAg. Furthermore, of the 31 patients with detectable HBV DNA by week 48, five were tested for ETV resistance and none was found.

### The VIRGIL study

The European network of excellence for Vigilance against Viral Resistance (VIRGIL) performed a multicentre cohort study at over 10 European referral centres between 2005 and 2010 [23]. The cohort included 333 consecutive adult CHB patients treated with ETV monotherapy, of whom 243 were NUC-naïve. Among the NUC-naïve patients, the cumulative probability of achieving virologic response at week 144 was 90% in HBeAg(+) patients and 99% in HBeAg(−) patients, and the proportion of HBeAg(+) patients with HBeAg loss was 34%. Of the five patients experiencing virologic breakthrough, no mutations associated with decreased sensitivity to ETV were found, including in those patients with viral loads >200 IU/mL at the end of follow-up. Additionally, among the 36 patients with detectable viral load at week 48, 81% went on to achieve undetectable levels of HBV DNA after prolonged ETV therapy, suggesting that even patients who only achieve a partial virologic response initially can benefit from continued ETV therapy. A later analysis of data from this cohort, including a total of 372 patients, showed that the decline in HBV DNA over 144 weeks of ETV treatment was comparable among patients with no cirrhosis (*n* = 274) and those with cirrhosis (*n* = 89) or decompensated cirrhosis (*n* = 9) [24]. Virologic response to ETV treatment was associated with a significantly reduced probability of disease progression to HCC, hepatic decompensation or death, even in patients with cirrhosis at baseline.

### Argentinean cohort

A retrospective, multicentre study was conducted at five centres in Argentina and included 69 treatment-naive chronic HBV patients receiving ETV for an average of 110 weeks. At baseline, patients were 63% HBeAg(+), 16% cirrhotic, mean HBV DNA was 7.09 log IU/mL, and mean alanine transaminase (ALT) was 157 IU/mL. Virologic response was reached by 46%, 77% and 100% of patients at week 24, 48 and 96, respectively. In the HBeAg(+) population, 84% of patients achieved virologic response, with 67% and 100% response rates seen at week 48 and 96, respectively. Among HBeAg(−) patients, 96% achieved virologic response, with 91% and 100% response rates seen at week 48 and 96, respectively. Twenty-three patients (53%) cleared HBeAg with 19 (44%) demonstrating HBeAg seroconversion; seven patients (10%) cleared HBsAg with five (7%) demonstrating HBsAg seroconversion [25].

### King’s College Cohort

This single-centre cohort study included 406 treatment-naïve patients, 154 of whom were treated with ETV monotherapy for a median of 28 months [26]. At baseline, 60% of patients had HBV DNA >4 log_10_ IU/mL and 34% had cirrhosis. Median HBV DNA declined continuously over the treatment period; 76% of patients had HBV DNA <12 IU/mL by month 12, and this proportion appeared to remain stable through to month 28. HBeAg seroconversion was reported in 8% of patients and 1% cleared HBsAg.

### The Italian cohort

**Fig 1:**
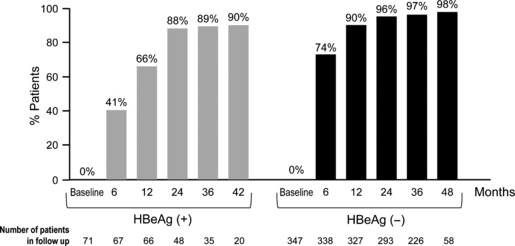
Virologic response by HBeAg status during entecavir (ETV) treatment in the Italian cohort real-life study. Percentage of ETV-treated patients achieving undetectable HBV DNA levels (<12 IU/mL) through 30 months of treatment in an Italian multicentre cohort study [26]. *Left*, HBeAg(+) patients; *right*, HBeAg(−) patients.

A retrospective/prospective, multicentre study was conducted at 19 Italian centres and included 418 consecutive NUC-naïve CHB patients initiating treatment with ETV [27]. Baseline characteristics differentiated this cohort from clinical trial patients in that they were predominately older (median age 58 years), genotype D HBV carriers (90%), 49% had cirrhosis, approximately 46% had a body mass index over 25 kg/m^2^, and 56% had concomitant diseases (Table 1). Despite these differences, treatment outcomes were similar to those found during Phase III clinical trials, with undetectable HBV DNA achieved by 85% of patients during the first year of treatment. Viral suppression reached 90% and 98% over 42 months of treatment in HBeAg(+) patients and 48 months of treatment in HBeAg(−) patients, respectively (Fig. 1). There were only three (1%) primary nonresponders (<1 log IU/mL drop in HBD DNA at week 12), and partial virologic responses (residual viraemia at week 48) were seen in 12% of patients. Virologic breakthrough was observed in 4% of patients over the full treatment duration. In HBeAg(+) patients, HBeAg seroconversion occurred in 27 patients (cumulative rate of 55%) and HBsAg loss in 12 patients (cumulative rate of 21%). No resistance to ETV was documented over the treatment period [27].

### The Hong Kong cohort

**Fig 2:**
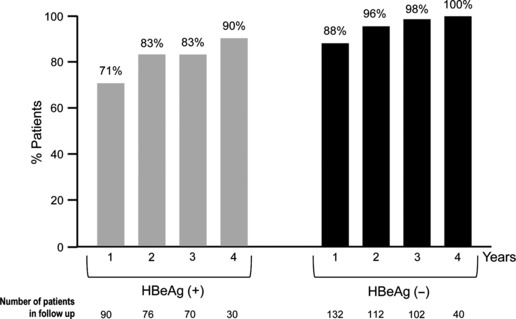
Virologic response by HBeAg status during entecavir (ETV) treatment in the Hong Kong cohort real-life study. Percentage of ETV-treated patients achieving undetectable HBV DNA levels (<12 IU/mL) through 4 years of treatment in the Hong Kong cohort [27]. *Left*, HBeAg(+) patients; *right*, HBeAg(−) patients.

The single-centre Hong Kong cohort study prospectively analysed 222 treatment-naïve patients receiving ETV (0.5 mg daily) for up to 4 years [28]. This long-term dosage with 0.5 mg ETV, the approved dosage for NUC-naïve patients, differentiates this study from the long-term ETV-901 clinical trial study, which doubled ETV dosage to 1.0 mg after year 1. The cumulative rate of achieving virologic response was 96% by year 4 (Fig. 2), with 86.4% and 100% of patients having HBV DNA >8 log_10_ copies/mL or <8 log_10_ copies/mL at baseline achieving undetectable levels of HBV DNA by the end of the therapy, respectively. Only one case of resistance (equating to a 0.6% cumulative resistance rate up to year 4) was reported in this patient cohort, as is consistent with clinical trial findings. HBeAg seroconversion occurred in 53%, but only one case of HBsAg seroconversion (0.5%) was reported; this low rate, by comparison with the Italian results, probably evidences the less frequent occurrence of HBsAg loss (mainly if not exclusively reported in HBeAg(+) patients) in genotypes B and C (the most frequent in Asia) compared with genotypes A and D (the most frequent in Europe).

## Efficacy of Tenofovir in Real-Life Settings

### King’s College Cohort

This cohort study (already discussed above) included 60 patients receiving first-line TDF treatment (Table 3) [26]. As TDF was approved after ETV, patients in the TDF group initiated treatment more recently than those initiating ETV treatment. As a result, they had a shorter duration of treatment at the time of the analysis (9 months compared with 28 months) and appeared to have less advanced disease than patients receiving ETV; in the TDF group, significantly fewer patients were HBeAg(+) (23%*vs* 31%, *P* < 0.05), and significantly fewer patients had cirrhosis at baseline (23%*vs* 34%, *P* < 0.05). The decline in HBV DNA over 12 months of treatment was comparable between patients treated with TDF and those treated with ETV (80%*vs* 76%). HBeAg seroconversion was reported in 7% of patients; no patients cleared HBsAg.

**3:** Baseline characteristics of patients included in tenofovir studies

Characteristic, *n* (%)*	Study 103	Study 102	King’s College Cohort	European cohort
Reference	[16]	[16]	[25]	[29]
*N*	176	250	60	302
Age, years (range)	34 (11)†	44 (11)†	40‡	55 (19–80)‡
Male	119 (68)	193 (77)	30 (50)	223 (74)
Race				
White	92 (52)	161 (64)	NR	NR
Asian	64 (36)	63 (25)		
Black	13 (7)	8 (3)		
Other	7 (4)	18 (7)		
Region				
Europe	97 (55)	158 (63)	UK	Europe
North America	47 (27)	53 (21)		
South America				
Australia and Asia	32 (18)	39 (16)§		
Genotype D	55/173 (32)	156/243 (64)	NR	NR
HBeAg(−)	0	250 (100)	46 (77)	242 (80)
HBV DNA, log_10_ IU/mL*	8.64 (1.076)†,¶	6.86 (1.31)†,¶	4.2 (0.2)**	5.9 (1.4–>9)‡
ALT, IU/L	142 (102.81)†	127.5 (101.21)†	NR	NR
Cirrhosis	34/172 (20)	47/250 (19)	14 (23)	106 (35)

ALT, alanine transaminase; HBV, hepatitis B virus; NR, not reported.

* Unless otherwise specified.

† Mean (standard deviation).

‡ Median (range).

§ Australia or New Zealand.

¶ log_10_ copies/mL.

** Mean (standard error).

### The European cohort

**Fig 3:**
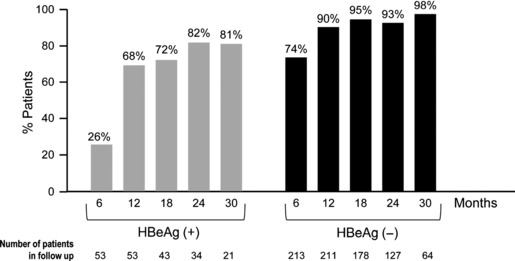
Virologic response by HBeAg status during tenofovir treatment in the European cohort real-life study. Percentage of tenofovir-treated patients achieving undetectable HBV DNA levels (<12 IU/mL) through 30 months of treatment in a European multicentre cohort study [29]. *Left*, HBeAg(+) patients; *right*, HBeAg(−) patients.

A multicentre cohort study, conducted at 19 European centres, retrospectively and prospectively monitored 302 consecutive NUC-naïve patients with CHB receiving TDF (245 mg) for a median of 28 months (range 0–60 months) [29]. Baseline characteristics differentiated these patients from Phase III clinical studies in that they were older, 35% had cirrhosis, and 45% had concomitant diseases (Table 3). Despite these differences, the efficacy of TDF was comparable to that observed in Phase III studies, with viral suppression (HBV DNA <12 IU/mL) being achieved by the majority of HBeAg(+) and HBeAg(−) patients by month 30 (Fig. 3). ALT normalization occurred in 100 (87%) patients by month 30. HBeAg seroconversion was seen in 11 patients and HBsAg loss was seen in seven patients. There were six (2.5%) primary nonresponders and 41 (15%) patients with partial virologic response (residual viraemia at week 48). Virologic breakthrough was reported in 2% of patients, with no potentially resistance-associated mutations identified to date.

## Safety and Tolerability of Entecavir in Real-Life Settings

Assessing how the safety and tolerability data from Phase III trials translate to those seen in general populations is obviously important as most patients will experience long-term or definitive therapies. Safety profiles in real-life studies of ETV in more than 1000 patients have been largely consistent with those of Phase III studies, in that no major safety issues or serious side effects have been reported to date [22–28]. Mitochondrial toxicity has been raised as a potential concern with NUCs as they can inhibit mitochondrial polymerase gamma, causing mitochondrial DNA depletion and subsequent mitochondrial toxicity. However, ETV has a low potential for interfering with polymerase gamma and causing mitochondrial toxicity [30]. This is consistent with a low incidence of adverse events attributable to mitochondrial toxicity reported in Phase III trials.

A consequence of mitochondrial toxicity can be lactic acidosis [31,32]. One retrospective study identified five cases of lactic acidosis among 16 ETV-treated patients with decompensated liver disease [31]. The five patients in question all had highly impaired liver function, with Model for End-Stage Liver Disease (MELD) scores of 22 or higher. In a prospective follow-up study in 37 patients with decompensated cirrhosis receiving LVD, ADV, ETV or TDF, four cases of lactic acidosis were reported, two in patients receiving TDF and two in untreated patients [32]. Furthermore, no cases of lactic acidosis were reported in the long-term rollover study ETV-901 [13]. Data from patients with hepatic decompensation also report few cases of lactic acidosis, with no cases recorded during 1 year of ETV therapy [33], none in the ETV/TDF/emtricitabine study [34] and one case in study ETV-048 (comparing ETV with ADV), which required no treatment and resolved despite continued ETV therapy [35]. As lactic acidosis appears to be observed more frequently in patients with decompensated disease, consideration should be given to monitoring these patients for this adverse effect regardless of the drug they are receiving. Carcinogenesis has been reported in animal models exposed to very high doses of ETV. However, to date, there is no evidence for the occurrence of cancers as a result of ETV treatment in patients. A global Phase IV study (the REALM study) is continuing to address this safety concern in patients treated with ETV during a 10-year follow-up period.

## Safety and Tolerability of Tenofovir in Real-Life Settings

Safety data collected from the European cohort study concerning TDF [29] were generally consistent with the long-term clinical study safety data. Nephrotoxicity may be a potential concern with TDF, based on evidence from post-marketing surveillance of patients receiving TDF for HIV infection [36], but so far the problem appears to be less evident in patients with HBV infection. In clinical trials in HBV-monoinfected patients, creatinine clearance rates remained stable over 4 years with <1% of patients having confirmed increases in creatinine levels of 0.5 mg/dL [17]. Mauss and colleagues [37] estimated the glomerular filtration rate (eGFR) in patients receiving TDF for HBV or HIV monoinfection using the Chronic Kidney Disease Epidemiology Collaboration (CKD-EPI) formula: predicted median changes in individual eGFR were much lower in HBV-infected patients (−0.92 mL/min) than in HIV-infected patients (−2.64 mL/min). In the European TDF cohort, no major changes in renal function were observed [29]. Increases in serum creatinine of >0.5 mg/dL were recorded in 1% of patients. The proportion of patients with an eGFR of <50 mL/min (as calculated by the Modification of Diet in Renal Disease formula) remained stable over the treatment duration; however, 6% of the patients had to stop or reduce TDF because of adverse events, which were renal-related in 4%– a number of these patients had baseline renal comorbidities, including hypertension and diabetes mellitus.

## Discussion

‘Real-life’ studies provide valuable information about the use of treatments in clinical practice, as they include patient populations usually under-represented in clinical studies and can identify rare or late-emerging adverse events. The importance of ‘real-life’ data is increasingly acknowledged, because in everyday practice, CHB patients are a heterogeneous population, often older, with a wide range of characteristics, morbidities, comorbidities and lifestyles. Moreover, many of the patients usually excluded from clinical trials, such as those with advanced liver disease, are those most in need of CHB therapy; thus, data to inform the management of these patients are of great relevance. The data presented in this review show that so far the real-life studies assessing ETV and TDF as first-line therapies for CHB have confirmed the results obtained in clinical trials, with both agents achieving response rates comparable to those in registration studies and with low rates of resistance development and favourable safety profiles. The importance of continued monitoring of safety in a clinical setting is highlighted by the case of TDF in the treatment of HIV, where cases of proximal tubular dysfunction were only observed post-marketing. The existence of multiple co-factors in HIV-infected patients, including combination therapy and the effects of the infection itself, precludes any extrapolation of these observations to the use of TDF in the treatment of HBV. However, careful monitoring is still recommended until a sufficient body of post-marketing experience had been acquired [36]. To avoid possible complications in patients with CHB receiving TDF long-term, it is advisable to identify any underlying risks for renal toxicity, including diabetes mellitus, atherosclerotic disease and older age – which are often excluded during clinical trials but will be present in real-life populations – and, as for all NUCs, to maintain dynamic dose adjustments according to creatinine clearance [2,36,38]. To this end, an algorithm for the assessment and follow-up of renal and bone abnormalities potentially associated with TDF has been recently proposed [36].

Real-life studies are limited by the fact that they often lack a standardized patient management protocol, are bound by a less stringent evaluation of efficacy and safety parameters, are prone to under-reporting of side effects and are often retrospective in nature. Moreover, smaller and less experienced investigation centres are often not included in real-life studies, which may result in a bias of the results towards higher response rates from the bigger, more experienced centres. Another caveat of real-life studies is that patient compliance to treatment regimens may potentially be poorer than in a clinical setting, which, in the case of CHB therapy, can compromise the response to treatment, and lead to virologic breakthrough and drug resistance. Interestingly, however, in real-life studies monitoring compliance to NUC therapies, poor adherence was less common than expected. In one study, nonadherence rates increased over time, yet remained below 10% throughout 4 years of treatment [39]. Another study found that over a median of 58 months, 61% of patients were totally adherent, and only 7% were nonadherent [40]. Another limitation of real-life studies is patient heterogeneity, which, although beneficial in terms of providing information on diverse populations, may confound data analyses if the data are pooled. For instance, in two studies that included investigation of renal safety, signs of proximal tubular damage were prevalent in 25–37% of patients treated with TDF [41,42]. These studies were confounded by the inclusion of both NUC-naïve and NUC-experienced patients, which may partly explain the difference to other TDF real-life studies [29] including only NUC-naïve patients.

Despite these limitations, the results of real-life studies provide an important addition to our knowledge of the effects of NUC therapy, and continued monitoring is warranted as a means of verifying the long-term efficacy and tolerability of these agents in a wide range of patients. In the real-life studies discussed in this review, including five studies assessing up to 4 years of treatment with ETV in more than 1000 patients and one study assessing up to 21 months of treatment with TDF in approximately 300 patients, ETV and TDF demonstrated long-lasting efficacies with minimal resistance rates, reversal of liver disease and favourable safety profiles, thus confirming the results reported in clinical trials.

## Abbreviations:

ADV: adefovir
ALT: alanine transaminase
CHB: chronic hepatitis B
eGFR: estimated the glomerular filtration rate
ETV: entecavir
HBeAg: hepatitis B e antigen
HBsAg: hepatitis B surface antigen
HBV: hepatitis B virus
HCC: hepatocellular carcinoma
LVD: lamivudine
NUCs: nucleos(t)ide analogues
TDF: tenofovir disoproxil fumarate
